# Repetitive Peroxide Exposure Reveals Pleiotropic Mitogen-Activated Protein Kinase Signaling Mechanisms

**DOI:** 10.1155/2011/636951

**Published:** 2010-12-19

**Authors:** Wayne Chadwick, Alex Keselman, Sung-Soo Park, Yu Zhou, Liyun Wang, Randall Brenneman, Bronwen Martin, Stuart Maudsley

**Affiliations:** ^1^Receptor Pharmacology Unit, Laboratory of Neuroscience, National Institute on Aging, National Institutes of Health, Baltimore, MD 21224, USA; ^2^Miller School of Medicine, University of Miami, Miami, FL 33136, USA; ^3^Metabolism Unit, Laboratory of Clinical Investigation, National Institute on Aging, National Institutes of Health, Baltimore, MD 21224, USA

## Abstract

Oxidative stressors such as hydrogen peroxide control the activation of many interconnected signaling systems and are implicated in neurodegenerative disease etiology. Application of hydrogen peroxide to PC12 cells activated multiple tyrosine kinases (c-Src, epidermal growth factor receptor (EGFR), and Pyk2) and the serine-threonine kinase ERK1/2. Peroxide-induced ERK1/2 activation was sensitive to intracellular calcium chelation and EGFR and c-Src kinase inhibition. Acute application and removal of peroxide allowed ERK1/2 activity levels to rapidly subside to basal serum-deprived levels. Using this protocol, we demonstrated that ERK1/2 activation tachyphylaxis developed upon repeated peroxide exposures. This tachyphylaxis was independent of c-Src/Pyk2 tyrosine phosphorylation but was associated with a progressive reduction of peroxide-induced EGFR tyrosine phosphorylation, EGFR interaction with growth factor receptor binding protein 2, and a redistribution of EGFR from the plasma membrane to the cytoplasm. Our data indicates that components of peroxide-induced ERK1/2 cascades are differentially affected by repeated exposures, indicating that oxidative signaling may be contextually variable.

## 1. Introduction

Our current knowledge of the complexity of cellular signaling systems is only now allowing us to appreciate the true depth of connectivity in what was previously thought to be collections of linear transduction cascades. It is clear from many lines of study that the structure of signaling systems is far more complex than first thought [1–4]. Cellular signaling networks underpin most of the biological systems that help maintain physiological systems. Intracellular signal transduction, often referred to as intermediary metabolism, can be controlled by the activation of cell surface receptors, alterations in ion flux through specific or nonspecific transmembrane channels, and also by small chemical molecules that are produced through enzymatic or chemosynthetic processes such as nitric oxide or hydrogen peroxide [5]. Other chemically minimal agents such as hydroxyl radicals or peroxynitrite have also been studied, with the latter recently attracting attention as a potential signaling molecule [6]. With respect to the involvement of these simple signaling agents, both nitric oxide and hydrogen peroxide have garnered the most interest. Nitric oxide signaling mechanisms have been well delineated and are primarily connected with the modulation of guanylyl cyclase-dependent processes [7–10]. In contrast to nitric oxide, reactive oxygen species (ROS), such as hydrogen peroxide, are known to regulate a multitude of cellular signaling and physiological processes. The generic term, ROS, includes species such as hydrogen peroxide (H_2_O_2_), the hydroxyl radical (^•^OH), superoxide (O_2_^−^), and singlet oxygen. ROS were originally recognized as active products produced from phagocytic neutrophils [11], which were thought to act as cytotoxic antimicrobial agents. A great deal of research upon ROS has therefore focused upon its association with tissue pathologies, for example, tumorigenesis and responses to hypoxia [12]. With respect to cancer signaling pathways, ROS are thought to contribute to aberrant cell growth through interference with multiple signaling systems involving nuclear transcription factor *κ*B, activated protein-1, phospholipase A_2_, and mitogen-activated protein kinases (MAPKs) such as the extracellular signal-regulated kinase (ERK), Akt, and Jun kinase [12].

Hydrogen peroxide is the major physiologically relevant form of ROS due to its relatively high stability. Many nonphagocytic cells are now known to produce H_2_O_2_ in response to a variety of physiological stimuli such as cytokines, peptidergic growth factors and neurotransmitters [13–16]. Liberated H_2_O_2_, even at subtoxic concentrations, affects the function of various proteins including transcription factors, phospholipases, protein kinases and phosphatases, ion channels, G proteins, G protein-coupled receptors, and receptor tyrosine kinases [16–20]. One of the primary signaling actions that may associate both the physiological and pathophysiological actions of H_2_O_2_ is the control of cellular tyrosine phosphorylation. H_2_O_2_ plays a key role in reversible protein phosphorylation through modulation of protein-tyrosine phosphatases, the lipid phosphatase, and tumor suppressor PTEN as well as multiple receptor tyrosine kinases, potentially through direct oxidation of their catalytic cysteine residues [21–24]. H_2_O_2_ therefore has been recognized as an important signaling mediator in growth factor functionality and more specifically, cell signaling-regulated phosphotyrosine posttranslational modification [23]. In many *in vitro* experiments, such as those mentioned previously, the signaling effects of H_2_O_2_ are usually explored in the context of persistent and chronic exposure to H_2_O_2_. As the generation and functioning of H_2_O_2_ may be controlled by humoral signaling systems in physiological settings, it is highly likely that, *in vivo,* the presence of H_2_O_2_ may vary from time to time according to physiological neurotransmission or hormonal release patterns. To investigate the effects of intermittent H_2_O_2_ exposure, we studied the effects of repetitive H_2_O_2_ exposure to neural PC12 cells with regards to cellular tyrosine kinase and ERK-activation properties. Both singular and repeated exposure of PC12 cells to H_2_O_2_ resulted in rapid increases in whole-cell protein tyrosine phosphorylation, specific phosphotyrosine content increases in receptor and nonreceptor tyrosine kinases and the potent activation of ERK. Repeated exposures to H_2_O_2_, however, resulted in the differential tachyphylaxis of specific components of the peroxide-dependent ERK-activation mechanisms. Such evidence may suggest that, *in vivo*, a complex interplay between multiple H_2_O_2_-controlled signaling processes may underlie its multiple physiological activities.

## 2. Materials and Methods

### 2.1. Chemicals and Reagents

Cell culture grade hydrogen peroxide, monodansylcadaverine (MDC), methyl-*β*-cyclodextrin (Me*β*C), sucrose, filipin, and the protein kinase A inhibitor H-89 were obtained from Sigma-Aldrich (St. Louis, MO). PP2 (non-receptor Src-family tyrosine kinase inhibitor), wortmannin (phosphoinositide 3-kinase inhibitor), BAPTA-AM (cell-permeable calcium chelator), PD98059 (MEK1/2 inhibitor) and AG1478 (epidermal growth factor receptor kinase inhibitor) were all obtained from EMD Bioscience (Gibbstown, NJ). Qproteome subcellular compartmentalization purification kits were obtained from Qiagen (Qiagen, Valencia, CA). The generic anti-phosphotyrosine antisera, PY99, anti-epidermal growth factor (EGF), anti-actin, anti-catalase, non-phosphorylated ERK1/2, anti-Grb2, anti-epidermal growth factor receptor, anti-lamin A, anti-Tim23 and anti-annexin V antisera were all obtained from Santa Cruz Biotechnology (Santa Cruz, CA). An agarose-preconjugated PY20 (anti-phosphotyrosine) slurry was also obtained from Santa Cruz Biotechnology. Anti-phosphosite-specific anti-Pyk2 (anti-phospho-Tyr402/Tyr881) and anti-c-Src (anti-phospho-Tyr418) were obtained from Invitrogen (Carlsbad, CA) and BD Bioscience respectively. Anti-active, (phosphor-)ERK1/2 antisera were obtained from Cell Signaling Technology (Danvers, MA). Non-phosphorylated anti-Pyk2 antisera were obtained from BD Bioscience.

### 2.2. Cell Culture

Rat pheochromacytoma cells (PC12), obtained from the American Type Tissue Culture Collection (http://www.atcc.org/), were maintained as previously described [3]. Briefly, PC12 cells were maintained in RPMI 1640 media supplemented with 10% horse serum, 5% fetal bovine serum, and 1% antibiotic/antimycotic (penicillin/streptomycin/fungizone: Invitrogen, Carlsbad CA) at 37°C in a 5% CO_2_ atmosphere. Prior to any cellular challenges with hydrogen peroxide, the cells were serum deprived for 16 h by removing the horse and bovine serum content of the RPMI growth medium and addition of 10 mM HEPES to compensate for the reduced buffering capacity caused by serum withdrawal. All PC12 cells were maintained in 100-mm plates coated with poly-d-lysine (Sigma Aldrich, St. Louis, MO). To experimentally degrade the exogenously-applied hydrogen peroxide, bovine liver catalase (Sigma Aldrich, St. Louis, MO) was added to the experimental media at a concentration of 40 U/mL. Bovine liver catalase was inactivated with a 95°C incubation for 10 minutes.

### 2.3. Immunoprecipitation and Western Blotting

After challenges with hydrogen peroxide, PC12 cell monolayers were placed on ice, washed twice in ice-cold Dulbecco's phosphate-buffered saline, and lysed in a Nonidet P-40 (NP40-)based solubilization buffer, as described previously [25]. Solubilized lysates were clarified by centrifugation at 15,000 rpm for 15 min and diluted to an approximate concentration of 1 mg/mL total protein. Subsequently, a 50 *μ*L aliquot of clarified whole-cell lysate was mixed with an equal volume of 2× Laemmli sample buffer and resolved by SDS-PAGE for determination of intracellular protein activation by immunoblotting. Immunoprecipitation (IP) of generic phosphotyrosine proteins was achieved using 20 *μ*L of a 50% slurry of anti-PY20 affinity agarose (Santa Cruz Biotechnology) with agitation for 16 h at 4°C. Immunoprecipitation of Pyk2 or EGFR was achieved using the addition of 5 *μ*g of the specific anti-protein sera plus 30 *μ*L of a protein-A/G-conjugated agarose slurry (EMD Bioscience) followed by agitation for 16 h at 4°C. Immune complexes were washed three times with ice-cold NP40-based lysis buffer and transferred to a clean microcentrifuge tube before addition of 20 *μ*L of 2× Laemmli sample buffer. Immunoprecipitates were resolved by SDS-PAGE and electrotransferred to polyvinylidene difluoride (PerkinElmer Life Sciences: Waltham, MA) membrane for protein immunoblotting (IB). Polyvinylidene difluoride membranes were blocked in a bovine serum albumin-based solution (4% bovine serum albumin, 50 mM Tris-HCl, pH 7.0, 0.05% Tween 20, 0.05% NP40) solution for 1 h at 37°C before immunoblotting. Immunoblotting of whole-cell lysates for unphosphorylated/phosphorylated ERK1/2 or inactive/active c-Src (Tyr-418 autophosphorylated) was performed using specific primary antisera (see under “*Chemicals and Reagents*”) at a 1 : 1000 dilution with subsequent addition of a 1 : 7000 dilution of alkaline phosphatase-conjugated anti-mouse or rabbit IgG as a secondary antibody (Sigma Aldrich). Immunoblotting of anti-phosphotyrosine immunoprecipitates, resolved with SDS-PAGE, for phosphotyrosine content was performed using a 1 : 1000 dilution of PY99 antisera with a subsequent addition of a 1 : 7000 dilution of alkaline phosphatase-conjugated anti-mouse IgG as a secondary antibody (Sigma). Immunoblotting of anti-Pyk2 or EGFR immunoprecipitates was performed using either anti-PY20 (1 : 1000 dilution for primary followed by 1 : 7000 dilution of alkaline phosphatase-conjugated anti-mouse secondary antisera), anti-phosphosite-specific (Tyr-402 or Tyr-881) anti-Pyk2 antisera (1 : 1000 dilution followed by incubation with a 1 : 7000 dilution of an alkaline phosphatase-conjugated anti-mouse secondary antisera) or with anti-Grb2 antisera (1 : 1000 primary antisera dilution followed by a 1 : 7000 dilution of alkaline phosphatase antirabbit-conjugated secondary antisera). Specifically isolated Qproteome cell compartments (CE1-cytoplasmic; CE-2 plasma membrane; CE-3 nuclear) were identified using 1 : 1000 dilutions of specific primary antisera, CE1-annexin-V, CE2-Tim23, or CE3-lamin-A followed by 1 : 7000 dilutions of species-specific alkaline phosphatase-conjugated secondary antisera (lamin A-mouse; Tim23-goat; annexin V-mouse). Visualization of alkaline phosphatase-labeled proteins was performed using enzyme-linked chemifluorescence (GE Healthcare-Amersham Biosciences) and quantified using a Typhoon 9410 PhosphorImager (GE Healthcare). Image density measurements were performed from phosphorimager-captured images using L-Process version 2.2 and Image-Gauge version 4.2 (Fuji Image Systems). Western blot image density was measured per square visual pixel (px^2^) as arbitrary units (AUs) from which background (B) signals were subtracted. Therefore western image density is represented throughout as (AU-B)/px^2^. Statistical analysis of relative protein expression values was performed using an unpaired Student's *t*-test in GraphPad Prism version 5.0.

## 3. Results

### 3.1. Hydrogen Peroxide Exposure Induces Widespread Changes in Generic Cellular Protein Tyrosine Phosphorylation Status

**Figure 1:**
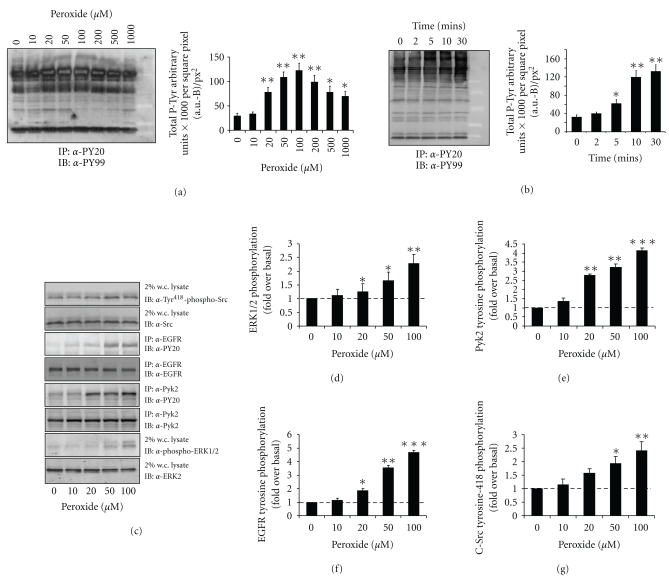
Hydrogen peroxide exposure generates increases in tyrosine phosphorylation status and activation of multiple signaling proteins in PC12 cells. (a) Hydrogen peroxide dose-dependent (10–1000 *μ*M) increases in whole-cell protein tyrosine phosphorylation. Hydrogen peroxide exposure time was 10 minutes. Total tyrosine phosphoproteins were purified by immunoprecipitation (IP) from PC12 cells using anti-phosphotyrosine antisera (PY20). Immunoblotting (IB) detection of the phosphotyrosine was achieved with an antisera raised against a differential phosphotyrosine immunogen (PY99). The associated histogram depicts the whole-lane image tyrosine phosphoprotein density quantitation measured as AU-B/px^2^. (b) Time-dependent hydrogen peroxide (100 *μ*M) increases in whole-cell protein tyrosine phosphorylation. The associated histogram depicts the whole-lane image tyrosine phosphoprotein density quantitation measured as AU-B/px^2^. (c) Hydrogen peroxide (10 minutes) exposure induces the activation of extracellular signal-regulated kinase (ERK1/2: detected in 2% of the total protein from a whole-cell (w.c.) lysate), tyrosine phosphorylation of Pyk2 and the epidermal growth factor receptor (EGFR) and activation of the non-receptor tyrosine kinase c-Src. (d), (e), (f), and (g) depict the peroxide-induced fold changes in phosphorylation of c-Src, EGFR, Pyk2, and ERK1/2, respectively. Values in each histogram depict the mean ± standard error for three individual experiments for each set of bars. For statistical analysis probability values indicated are *P < .05, **P < .01, and ***P < .001.

Short term application (10 minutes) of hydrogen peroxide to serum-deprived PC12 cells resulted in the dose-dependent increase in the generic phosphotyrosine content of multiple proteins identified using poylacrylamide gel resolution of anti-phosphotyrosine (PY20) immunoprecipitates derived from cell lysates. The dose response relationship, however, was not perfectly sigmoidal, as at peroxide concentrations greater than 200 *μ*M a lack of further phosphotyrosine increase was seen (Figure 1(a)). Employing the hydrogen peroxide concentration that yielded the most robust increases in whole-cell protein phosphotyrosine content (100 *μ*M), it was demonstrated that this effect was achieved and reached its maximum after 10 minutes of acute stimulation (Figure 1(b)). This time period of acute stimulation was therefore employed as the standard stimulation time in all subsequent experiments.

### 3.2. Acute Hydrogen Peroxide Exposure Induces a Dose-Dependent Increase of the Phosphorylation Status of Serine/Threonine and Tyrosine Kinases

Acute (10 minute) application of multiple doses (10–100 *μ*M) of hydrogen peroxide resulted in the significant increase of the levels of tyrosine-418 phosphorylation of the non-receptor tyrosine kinase c-Src (Figures 1(c) and 1(d)). This tyrosine phosphorylation site on c-Src occurs in the kinase activation region of the Src family kinases and is highly indicative of auto-tyrosine phosphorylation mediated by the intrinsic Src kinase activity itself. Significant dose-dependent increases in the phosphotyrosine status of the immunoprecipitated epidermal growth factor receptor (EGFR) were also noted in response to the acute hydrogen peroxide stimulation (Figures 1(c) and 1(e)). In an analogous manner to c-Src, upon activation EGFRs typically auto-tyrosine phosphorylate at multiple residues. This phosphorylation event is highly indicative of activation of the EGFR intrinsic tyrosine kinase. To assess whether any residual EGF (from initial serum-containing media used for prior cell growth) was present during the peroxide exposure, specific Western blot analysis was performed on nonconcentrated and SpeedVac-concentrated serum-deprivation media. We found that no extracellular EGF ligand was present in the serum-deprivation media during the peroxide exposures (data not shown). In addition to increases in the phosphotyrosine content of c-Src and the EGFR, we also noted a significant peroxide dose-dependent increase in the phosphotyrosine content of Pyk2, isolated using specific anti-Pyk2 immunopurification (Figures 1(c) and 1(f)). It has been demonstrated that all of these tyrosine kinase systems, c-Src, EGFR, and Pyk2, are similarly linked to conserved cellular signaling mechanisms that directly control the activation of MAPKs such as the serine/threonine targeting extracellular signal-regulated kinase (ERK1/2). When we assessed the activity status of ERK1/2 in response to the peroxide exposure, we noted a significant elevation in the activity status of ERK1/2 (Figures 1(c) and 1(d)) that coincided with the peroxide-induced increases in phosphotyrosine content of Pyk2 and the EGFR as well as the activity status of c-Src. 

### 3.3. Acute Removal of Applied Hydrogen Peroxide Attenuates Long-Term ERK Activation in PC12 Cells

**Figure 2:**
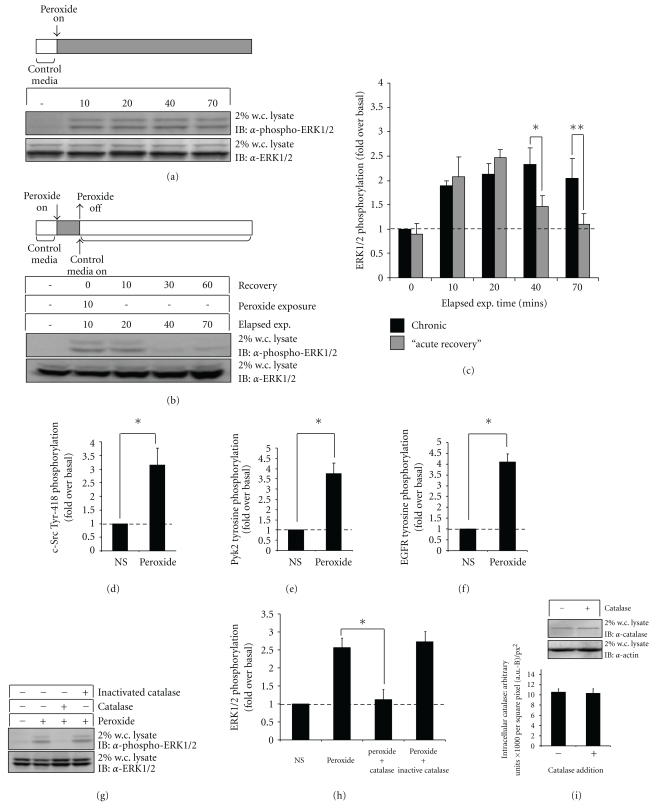
ERK1/2 responses to chronic or acute-recovery exposure to hydrogen peroxide. (a) Chronic hydrogen peroxide exposure (70-minutes, 100 *μ*M) and ERK1/2 activation (white bar-no peroxide, grey bar-peroxide exposure). (b) Acute-recovery hydrogen peroxide exposure (100 *μ*M) and ERK1/2 activation. (c) Chronic (black bars) versus acute-recovery (grey bars) peroxide exposure ERK1/2 activation profile. Acute-recovery peroxide exposure-induced activation of c-Src (d), Pyk2 (e) and EGFR (f) tyrosine phosphorylation. (g)-(h) Catalase (40 U/mL) (active or heat-inactivated) effects upon peroxide-induced (acute-recovery protocol) ERK1/2 activation. (i) Intracellular catalase levels following extracellular enzyme exposure.

As we noted that acute application of hydrogen peroxide to PC12 cells resulted in the simultaneous activation of multiple tyrosine kinase systems as well as ERK1/2, we investigated the ability of the PC12 cells to recover from such acute exposures. During neurological processes in aging, or during periods of stress, it is possible that cellular damage may be induced by both intermittent as well as chronic and protracted oxidative episodes. Exposure to long-term stresses tends to induce pathophysiologies and even cell death mechanisms whereas short-term exposures are more survivable [26, 27]. Application of 100 *μ*M hydrogen peroxide for at least 60 minutes resulted in a long-lasting elevation of the ERK1/2 activity status in the PC12 cells (Figure 2(a)). To simulate a short-term, survivable exposure to the peroxide, we performed parallel experiments to compare peroxide exposure paradigms that involved rapid acute exposure versus long-term exposure to the oxidative agent. Using multiple parallel experiments, we were able to simultaneously generate PC12 protein extracts at any time point during the experiment. We therefore applied the 100 *μ*M hydrogen peroxide dose for 10 minutes and then removed the peroxide-containing media and replaced it with media similar to that prior to the peroxide exposure (Figure 2(b)) to achieve an “acute-recovery” process. PC12 cell lysates were then extracted from plates that were naive to peroxide, directly at the end of the 10 minute peroxide exposure and then 10, 30, and 60 minutes after the removal of the peroxide exposure (recovery) (Figure 2(b)). We additionally performed control experiments to assess any effects of fluid replacement upon ERK1/2 activity in the PC12 cells. When performing multiple fluid exchanges, we did not notice any significant alterations in the PC12 ERK1/2 activity status in our cells (data not shown). We noted that the degree of ERK1/2 activation after the “acute-recovery” peroxide paradigm waned rapidly to nearly background levels at 30 minutes after the “acute-recovery” peroxide stimulation (Figure 2(c)). Therefore after 30 minutes from the removal of the acutely applied peroxide, the ERK1/2 activity reached basal levels, while with sustained peroxide exposure the PC12 cells demonstrated a well-maintained high-activity status of ERK1/2. Using this “acute-recovery” peroxide stimulation procedure, that is, 10-minute peroxide application followed by peroxide removal and a 10 minute recovery before protein harvesting, we also assessed the changes in activity status of c-Src, Pyk2, and EGFR. In a similar manner to the standard peroxide application, without removal and recovery, we noted that the “acute-recovery” peroxide protocol was capable of inducing significant increases in the phosphorylation of c-Src Tyr-418 resolved from whole-cell lysates (Figure 2(d)). In addition, phosphotyrosine immunoblotting of Pyk2 (Figure 2(e)) or EGFR (Figure 2(f)) immunoprecipitates demonstrated that the “acute-recovery” peroxide paradigm induced significant elevations of the phosphotyrosine content of these tyrosine kinases. In addition to assessment of the signaling functionality of the “acute-recovery” peroxide exposure, we demonstrated that the primary activity of the peroxide was mediated through its extracellular effects, rather than intracellular permeation, with the application of exogenous catalase (Figures 2(g) and 2(h)). After addition of active bovine liver catalase (40 U/mL) to the cell media (30 minutes preincubation) prior to peroxide exposure, we noted a significant reduction in the ERK1/2 activation signal. Heat treatment (95°C, 10 minutes) of the catalase before its addition to the peroxide-containing media prevented the inhibition of ERK1/2 activation (Figures 2(g) and 2(h)). Specific Western blot analysis of whole-cell lysates incubated for 30 minutes with extracellular catalase failed to demonstrate any significant changes of intracellular catalase levels, suggesting that negligible catalase internalization had occurred (Figure 2(i)). Therefore, the majority of the rapid effects of catalase upon peroxide activity are likely to have occurred in the extracellular space.

### 3.4. Chemical Sensitivity of “Acute-Recovery” Peroxide-Induced ERK Activation

**Figure 3:**
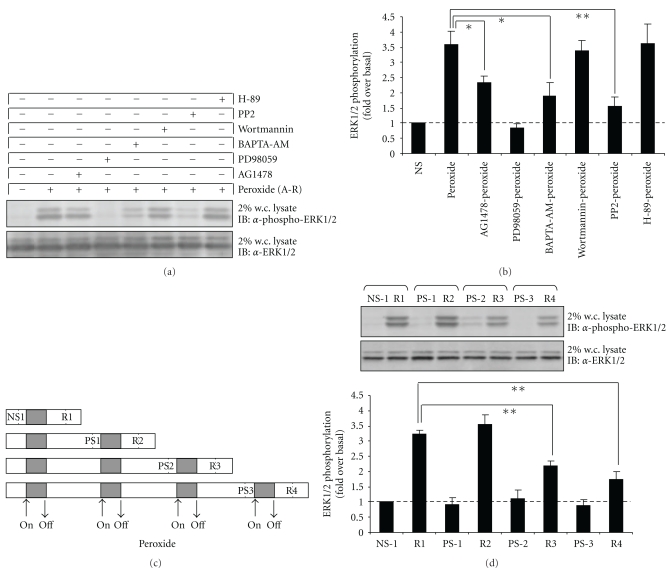
Chemical sensitivity and tachyphylaxis of the ERK activation response to the “acute-recovery” paradigm of hydrogen peroxide exposure. (a) Representative western blot of the chemical sensitivity of the acute-recovery paradigm peroxide-induced ERK activation (10 minute peroxide exposure, 10 minute recovery). Signaling reagents were pre-incubated with the PC12 cells prior to the “acute-recovery” peroxide exposure as follows: AG1478, 100 nM, 30 minutes; PD98059, 20 *μ*M, 60 minutes; BAPTA-AM, 50 *μ*M, 30 minutes; wortmannin, 10 nM, 30 minutes; PP2, 5 *μ*M, 30 minutes; H-89, 10 *μ*M, 30 minutes. (b) Histogram depicting the relative effects of the chemical pre-exposures from panel A to the fold over basal-induced ERK1/2 activation induced by the acute-recovery peroxide protocol. Each histogram bar depicts the mean ± standard error. (c) Diagrammatic representation of peroxide exposure procedures employed to derive protein extracts for examination of repeated peroxide exposure (grey panels) effects upon ERK1/2 activation (NS1—non-stimulated control ERK1/2 sample, R1—sample from 10 minutes after 10 minute acute-recovery peroxide process, PS2-PS3—prior-stimulation control levels before specific repeated peroxide exposure sample, R2–R4—repeated peroxide exposure protein samples). (d) The ERK1/2 activation responses to the repeated acute-recovery peroxide exposure demonstrates tachyphylaxis. The acute-recovery R1 response (10 minute exposure, 10 minute recovery) was followed by a 30 minute recovery before the same acute-recovery process was repeated, generating the R2 response. R3 and R4 responses were created in a similar manner from cells previously stimulated with R1, R2 and then subsequent R3 and R4 acute-recovery exposures. The associated histogram represents the mean ± standard error for three individual ERK1/2 R1-R4 tachyphylaxis experiments. For statistical analysis probability values indicated are *P < .05, **P < .01.

We next examined the potential mechanistic links between the tyrosine phosphorylation events induced by the “acute-recovery” peroxide exposure process and the ERK1/2 activation. Preincubation of PC12 cells with the specific EGFR kinase inhibitor AG1478, the c-Src kinase inhibitor PP2, and the Ca^2+^-chelator BAPTA-AM induced a partial inhibition of the ERK1/2 signal (Figures 3(a) and 3(b)). The inhibitory effects of BAPTA-AM were likely due to the chelation of extant intracellular calcium as our low peroxide dose employed (100 *μ*M) did not greatly elevate global calcium levels (data not shown). Direct inhibition of the upstream ERK1/2-phosphorylating kinase, MEK1/2, with PD98059 (20 *μ*M, 60 minute preincubation prior to peroxide exposure) resulted in near complete inhibition of the peroxide-induced ERK1/2 signal. The peroxide-induced ERK1/2 activation signal was, however, largely resistant to inhibition of phosphoinositide-3-kinase (PI-3K) or protein kinase A (PKA) as neither H-89 nor wortmannin preincubation affected the peroxide-induced ERK1/2 activation (Figures 3(a) and 3(b)). 

### 3.5. Acute-Recovery Peroxide Exposure Protocols Allow Assessment of Repeated Peroxide Exposure-Induced ERK1/2 Tachyphylaxis

 As the “acute-recovery” peroxide exposure paradigm allows the peroxide-induced ERK1/2 response to subside to basal levels within thirty minutes after peroxide removal, we performed multiple parallel experiments to analyze the ability of the PC12 cells to respond to multiple acute exposures. The pictogram in Figure 3(c) demonstrates the methodology of investigating the ability of the PC12 cells to respond to four successive peroxide exposures (all 100 *μ*M doses). To create protein samples for R1, the simple “acute-recovery” process was performed. To create protein samples for secondary (R2), tertiary (R3), and quaternary (R4) responses, individual “acute-recovery” peroxide applications were made and were separated by 30 minutes of recovery time (Figure 3(c)). When the levels of peroxide-induced ERK1/2 activation were measured for R1 to R4 responses it was noted that the degree of peroxide-induced ERK1/2 activation was significantly reduced by R3 and then again for R4. The greatest reduction in peroxide-induced ERK1/2 signal was observed for the R4 response, compared to R1. There was no significant diminution of the peroxide-induced ERK1/2 activation from R1 to R2 (Figure 3(d)). As the biggest changes in peroxide-induced ERK1/2 activation occurred at R4, we directly compared potential signaling mechanism differences between stimulation protocols for R1 samples compared to R4 samples.

### 3.6. Repeated Peroxide Exposure Causes Reduced EGFR Tyrosine Phosphorylation but Fails to Significantly Affect Pyk2 and c-Src Tyrosine Phosphorylation

**Figure 4:**
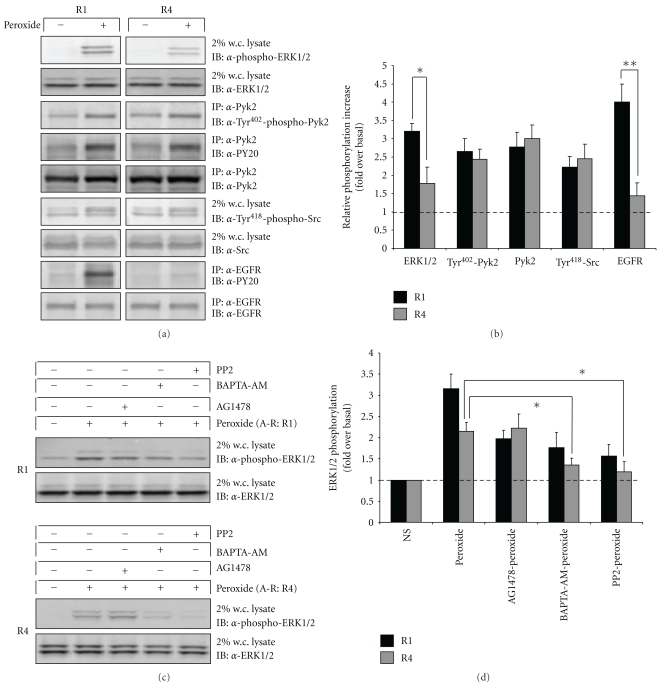
Repeated peroxide exposure differentially affects tyrosine kinase responses in PC12 cells. (a) Comparison of levels of peroxide-induced ERK1/2 activation, Pyk2 generic tyrosine and tyrosine-402 phosphorylation, c-Src tyrosine-418 phosphorylation and EGFR tyrosine phosphorylation between R1 and R4 responses. (b) The histogram depicts the quantitation of mean ± standard error for three individual experiments for the data represented in panel (a). The represented fold changes in ERK1/2, Pyk2 Tyr-402, Pyk2 generic tyrosine phosphorylation, c-Src Tyr-418 and EGFR tyrosine phosphorylation were calculated relative to the non-stimulated phosphorylation value prior to either R1 (black bars) or R4 (grey bars). (c) Chemical sensitivity of R1 versus R4 ERK1/2 phosphorylation responses to acute-recovery peroxide exposure paradigms. Preincubation times and chemical concentrations for AG1478, BAPTA-AM and PP2 were as used before in Figure 3. (d) The histogram depicts the quantitation of the mean ± standard error for three individual experiments for the data represented in panel (c) (R1-black bars; R4-grey bars). For statistical analysis probability values indicated are *P < .05, **P < .01.

As we have shown in the previous section, repeated peroxide “acute-recovery” exposures induce a partial tachyphylaxis of the ERK1/2 response. We then investigated the effects of repeated peroxide exposure upon peroxide-induced c-Src activation as well as peroxide-induced EGFR and Pyk2 tyrosine phosphorylation. Upon comparing the effects of repeated exposure to peroxide (R1 to R4), we noted that along with the expected reduction in ERK1/2 activation, at the R4 response point, the ability of the hydrogen peroxide to induce Pyk2 auto-tyrosine phosphorylation (indicative of Pyk2 tyrosine kinase activation—measured with anti-phospho-Tyr-402-Pyk2 sera) and increase generic Pyk2 tyrosine phosphorylation was not significantly affected (Figures 4(a) and 4(b)). As with the Pyk2 responses between R1 and R4 peroxide exposures, the degree of c-Src Tyr-418 phosphorylation was not significantly diminished by the repeated exposure (R1–R4) to the stressor (Figures 4(a) and 4(b)). In contrast, however, we noted that the degree of peroxide-induced EGFR tyrosine phosphorylation was significantly lower at the R4 response point compared to R1 (Figures 4(a) and 4(b)). We additionally assessed whether there were any differences in the signaling pathways to ERK1/2 used by the peroxide exposures at either R1 or R4. When determining the R1 effects we again noted that the peroxide-induced ERK1/2 response was partially sensitive to AG1478, BAPTA-AM, and PP2 preincubation (Figure 4(c)). When this R1-sensitivity was compared to the R4 response chemical sensitivity, we noted that the R4-mediated ERK1/2 responses were relatively insensitive to AG1478-mediated inhibition of the EGFR intrinsic tyrosine kinase (Figure 4(d)). The R4 responses, however, were still sensitive to the preincubation with BAPTA-AM and PP2 (Figure 4(d)).

### 3.7. Repeated Peroxide Exposure Attenuates EGFR Tyrosine Phosphorylation and Protein-Protein Interaction with Growth Factor Receptor Binding Protein-2

**Figure 5:**
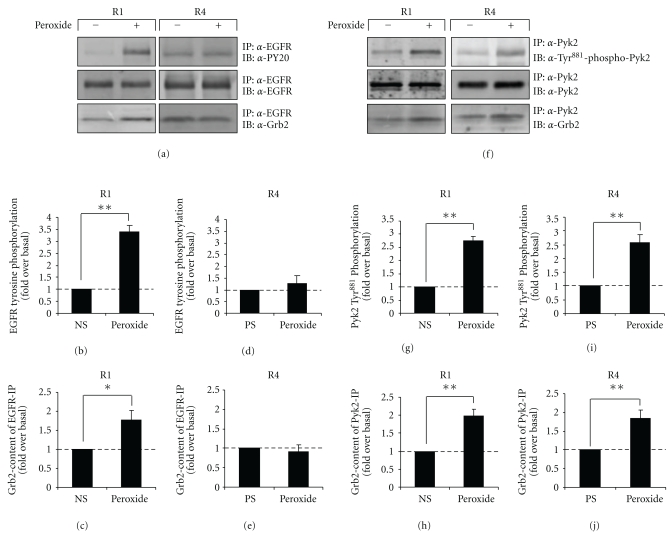
Grb2 association with EGFR or Pyk2 is differentially affected by repeated peroxide exposure. (a) Representative western blots of phosphotyrosine and Grb2 content of EGFR immunoprecipitates from cells stimulated to R1 or the R4 level with 100 *μ*M hydrogen peroxide. (b) EGFR immunoprecipitate tyrosine phosphorylation status in response to R1 hydrogen peroxide exposure (NS—non-stimulated). (c) Peroxide-induced fold changes in the Grb2 content of EGFR immunoprecipitates at the R1 response point. (d) EGFR immunoprecipitate tyrosine phosphorylation status in response to R4 hydrogen peroxide exposure (PS—pre-stimulated basal level prior to R4). (e) Peroxide-induced fold changes in the Grb2 content of EGFR immunoprecipitates at the R4 response point. (f) Representative western blots of Pyk2 Tyrosine 881 phosphorylation and Grb2 content of Pyk2 immunoprecipitates from cells stimulated to R1 or the R4 level with 100 *μ*M hydrogen peroxide. (g) Pyk2 immunoprecipitate tyrosine-881 phosphorylation status in response to R1 hydrogen peroxide exposure. (h) Peroxide-induced fold changes in the Grb2 content of Pyk2 immunoprecipitates at the R1 response point. (i) Pyk2 immunoprecipitate tyrosine-881 phosphorylation status in response to R4 hydrogen peroxide exposure. (j) Peroxide-induced fold changes in the Grb2 content of Pyk2 immunoprecipitates at the R4 response point. The histograms in each panel depict the quantitation of the mean ± standard error for three individual experiments. For statistical analysis *P < .05, **P < .01.

As we had noted that repeated exposures to peroxide reduced both the ERK1/2 activation and the degree of EGFR tyrosine phosphorylation, but not that of Pyk2 or c-Src, we decided to investigate whether additional signaling components of ERK1/2-activating complexes were affected. The growth factor receptor binding protein 2 (Grb2) is a bifunctional adapter protein involved in multiple ERK-activating multiprotein complexes. Grb2 is typically associated with Ras-guanine nucleotide exchange factors such as son of sevenless (Sos). Upon activation of scaffolding molecules employed in ERK1/2 activation cascades, for example, EGFR, Pyk2, or the focal adhesion kinase (FAK), the auto-tyrosine phosphorylation of these proteins attracts Grb2 to the plasma membrane via interaction of its SH2 domains with phosphotyrosine residues on the scaffolds. Grb2 interacts with Sos via its SH3 domains associating with proline-rich regions of Sos. At the plasma membrane, Sos can then facilitate exchange of guanosine triphosphate for guanosine diphosphate at juxtamembrane monomeric G proteins such as Ras. As Grb2 association with scaffolds, such as EGFR or Pyk2, is vital for their capacity to activate ERK1/2 cascades, we investigated the relative effects of R1- versus R4-level peroxide exposures upon Grb2 interaction with EGFR or Pyk2. As we have seen previously, successive peroxide exposures reduced the degree of peroxide-induced EGFR tyrosine phosphorylation (Figures 5(a) and 5(b)). When the Grb2 content of the EGFR immunoprecipitates was assessed, we noted that for R1 responses to peroxide exposure there was a significant increase in the Grb2 content of the purified EGFR (Figures 5(a) and 5(c)). With repeated peroxide exposures (R4), the degree of peroxide-induced EGFR tyrosine phosphorylation was attenuated (Figures 5(a) and 5(d)) along with a significantly reduced Grb2 content of the EGFR immunoprecipitates (Figures 5(a) and 5(e)). We have shown previously that peroxide stimulation increases the generic and Tyr-402-specific tyrosine phosphorylation of Pyk2 at R1 and R4 response points (Figure 4(a)). With respect to association with Grb2, phosphorylation of the Pyk2 tyrosine residue 881 has been shown to be critical [28]. Using specific antisera that recognizes tyrosine phosphorylation of residue Tyr-881 on Pyk2, we noted that at the R1 and R4 time points peroxide increased the phosphotyrosine content of this residue (Figure 5(f)). At the R1 response point, peroxide exposure induced a significant increase in Pyk2 Tyr-881 phosphorylation status (Figures 5(f) and 5(g)) along with a significant increase in the Grb2 content of the Pyk2 immunoprecipitate (Figures 5(f) and 5(h)). Unlike the tyrosine phosphorylation and Grb2 interaction with the EGFR, repeated peroxide exposure (at R4 response point) did not significantly affect Pyk2 Tyr-881 phosphorylation (Figures 5(f) and 5(i)) or the Grb2 content of the Pyk2 immunoprecipitates (Figures 5(f) and 5(j)). While Grb2 was observed to increase in its association with either EGFR or Pyk2 upon peroxide exposure, we also tested the possibility that a multimolecular complex containing all three proteins may also be induced. When EGFR immunoprecipitates were probed for the presence of Pyk2 (in the presence or absence of peroxide exposure), we failed to find any significant coassociation of these two proteins. When the converse western blot was performed, no significant EGFR content of Pyk2 immunoprecipitates was also demonstrated in the absence or presence of peroxide (data not shown). Therefore, it seems likely that peroxide treatment induces the creation of largely discrete Grb2-interacting EGFR or Pyk2 complexes.

### 3.8. Repeated Peroxide Exposure Induces a Selective Subcellular Redistribution of ERK1/2-Activating Scaffold Proteins

**Figure 6:**
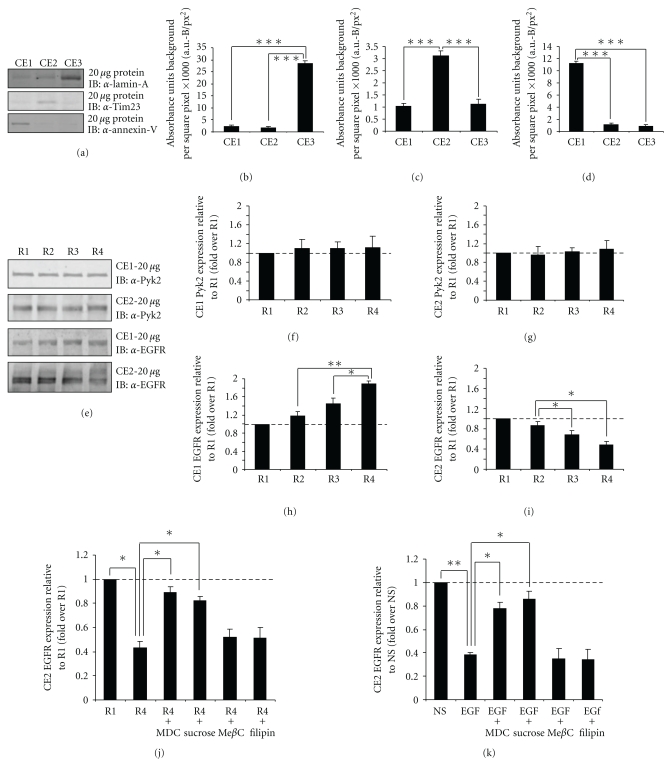
Peroxide-mediated subcellular relocalization of EGFR. (a) Subcellular fractionation verification (nuclear, lamin-A: CE3; plasma membrane, Tim23: CE2; cytosol, annexin-V: CE1) and quantitation ((b)-lamin-A, (c)-Tim23, (d)-annexin-V). (e) Peroxide effects (responses R1-R4) on Pyk2 and EGFR subcellular localization Relative expression profiles for Pyk2 (CE1-CE2 quantification (f)-(g)) and EGFR (CE1-CE2 quantification (h)-(i)). Chemical inhibitor-mediated alteration of R4 peroxide-mediated (j) or EGF (k) induced EGFR redistribution from CE2 fraction. EGF (10 ng/mL)-mediated redistribution was compared to non-stimulated (NS) cells.

It has been demonstrated by many researchers that the molecular and biochemical nature of a specific ERK1/2-activating scaffold has profound effects upon the physiological mechanisms of ERK1/2 signaling [4, 25]. To this end, we sought to investigate a potential difference in the ERK1/2-activating scaffolds in our study, EGFR and Pyk2, with respect to their abilities to respond to repeated peroxide exposure. The ability of many G protein-coupled receptors (GPCRs) to activate ERK1/2 has been shown to involve a productive engagement with classical endocytic processes [4]. This interaction has resulted in the demonstration of endocytic-dependent ERK1/2-activation processes. Rather than residing with the importance of endocytosis of the GPCR, it appears in many cases that the specific endocytic process interaction of the scaffold/Ras-activating machinery is more important. We therefore assessed whether the inhibitory actions of repeated peroxide exposure upon EGFR-associated ERK1/2 signaling may be related to a subcellular redistribution of the EGFR compared to Pyk2. Using a differential detergent-based cell compartmentalization process (Qproteome), we were able to successfully isolate subcellular fractions that demonstrated enrichment of nuclear proteins (lamin A-CE3), plasma membrane proteins (Tim23-CE2), and cytoplasmic proteins (annexin V-CE1) (Figures 6(a)–6(d)). Stimulation of cell surface receptors that are able to engage classical endocytic pathways often result in a rapid (10–30 minutes) redistribution from the plasma membrane to small early endocytic vesicles in the cytoplasm. When we applied successive peroxide exposures (R1–R4) and then isolated either the cytoplasmic (CE1) or plasma membrane (CE2) subcellular fractions, we noted that there was little alterations in the expression profile of Pyk2 across R1-R4 (Figures 6(e), 6(f), and 6(g)). In contrast, with successive peroxide exposures (R1–R4), we noted a significant increase in the amount of EGFR found in cytoplasmic extracts (Figures 6(e) and 6(h)), while a concomitant significant decrease of the EGFR found in plasma membrane extracts was observed (Figures 6(e) and 6(i)). Therefore it appeared that while Pyk2 expression levels did not significantly alter in the two compartments, there seemed to be a progressive relocalization of EGFR from the plasma membrane to the cytoplasm after repeated peroxide exposures. We additionally contrasted the nature of the peroxide-induced EGFR cellular redistribution process from the CE2 fraction using chemical inhibitors of two different forms of endocytic mechanisms, that is, clathrin or caveolae mediated [29]. Inhibition of clathrin-mediated endocytosis (30-minute preincubation before R4 peroxide exposure with 400 *μ*M MDC or hypertonic 0.45 M sucrose) significantly attenuated the peroxide-induced EGFR redistribution (Figure 6(j)). Inhibition of caveolae-mediated endocytic mechanisms (30-minute preincubation with Me*β*C (10 *μ*M) or filipin (5 *μ*g/mL)) failed to alter R4 peroxide-induced EGFR redistribution (Figure 6(j)). When the chemical sensitivity of EGF-induced (10 ng/mL, 30 minute exposure) redistribution of EGFR from CE2 was analogized to that mediated by peroxide, we noted a similar inhibitory effect of clathrin-mediated endocytosis disruptors (MDC, sucrose) and relative independence from caveolae-mediated mechanisms (Figure 6(k)). These data suggest therefore that the peroxide-induced redistribution of EGFR occurs through a qualitatively similar process to that of EGF-induced EGFR internalization. In a similar manner to attenuation of ERK1/2 signaling upon repeated peroxide exposure, we also observed a similar progressive reduction in EGF-induced ERK1/2 phosphorylation (% ERK1/2 phosphorylation compared to non-stimulated control: EGF exposure 1, 20.5 ± 1.3%; EGF exposure 2, 5.7 ± 0.9%; EGF exposure 3, 1.19 ± 1.03%; EGF exposure 4, 1.06 ± 0.87% (*n* = 3)) using a repeated exposure paradigm analogous to the peroxide stimulations. As both repeated peroxide and EGF mediated an effective redistribution of EGFR from the plasma membrane fraction, we also found that exogenous EGF application (10 ng/mL, 30 minutes) was able to partially blunt the subsequent ERK1/2 response to peroxide stimulation (% peroxide-induced ERK1/2 phosphorylation after EGF treatment, compared to non-EGF pre-exposed control, was 52 ± 5.7% (*n* = 3)). Therefore, it seems that the peroxide-mediated effects on the EGFR are similar in several ways to the normal functioning of the EGF/EGFR system.

## 4. Discussion

In this study, we have shown that in response to acute exposure of PC12 cells to hydrogen peroxide, multiple ERK1/2-activating mechanisms can be stimulated. We demonstrated that hydrogen peroxide exposure resulted in the elevation of the phosphotyrosine status of the non-receptor tyrosine kinase c-Src as well as the EGFR and Pyk2. Multiple studies have demonstrated that alterations in cellular oxidative state are a potent modulator of multiple diverse and interconnected intermediary cell metabolism signaling mechanisms [13, 30, 31]. Tyrosine kinase signaling systems are typically associated with the creation of multiprotein complexes. These complexes are involved in the control of growth factor or nuclear hormone receptor activity that subsequently stimulates MAPK pathways, which are strongly linked to long-term genotropic hormonal effects, such as cell survival or stress resistance [25, 32–34]. The intersection between tyrosine kinase signaling systems and peroxide-mediated signaling also appears to strongly regulate nongenotropic actions of mitogen-activated protein kinases (formerly known as microtubule-associated protein kinases), for example, cytoskeletal remodeling and adherence control [23, 35, 36]. One of the important features common to the tyrosine kinase factors we have identified to be activated in our PC12 cells by peroxide, that is, c-Src, EGFR, and Pyk2, is their ability to act as scaffolding proteins for MAPK-activating complexes [25, 37–40]. While all of these proteins are diverse in their nature, for example, EGFR activation requires dimerization and internalization to induce activation of many downstream effectors, Pyk2 activation often appears calcium dependent, and c-Src requires disruption of stable intramolecular interactions to become fully active [25, 41–45], they all demonstrate an important requirement for auto-tyrosine phosphorylation. With regards to this, we demonstrated that in each case, peroxide exposure was able to induce this posttranslational modification (Figures 1(c) and 4(a)). Therefore the action of peroxide in this instance is highly reminiscent, with regards to cell signaling, of the activation of cell surface receptors linked to these signaling pathways. This posit was reinforced by our finding that catalase-mediated degradation inhibited the ability of the exogenous hydrogen peroxide to activate ERK1/2 and tyrosine kinase systems (Figure 2(g)) [46, 47]. All of the peroxide-stimulated signaling factors we identified (c-Src, EGFR, Pyk2) have been strongly implicated as intermediaries in receptor-mediated MAPK/ERK1/2 activation [25, 47–50], again suggesting a reasonable analogy between the effects of exogenous peroxide and receptor-biological-like activity. Another important aspect of receptor-mediated activity is the controllable response to cell stimulation by extracellular ligands. Most receptor-based systems demonstrate rapid tachyphylaxis to the presence of excessive or repeated exposure to stimulating ligands [51, 52]. In recent years, the complexity of these regulatory mechanisms and their ability to modulate the signaling nature of the stimulus have been partially revealed. The receptor desensitization mechanisms are now considered to function as “conditioners” of the stimulating signal, rather than just blunt attenuators of the signal [38, 53–55]. Using our “acute-recovery” peroxide stimulation protocol, we were able to investigate the capacity of PC12 cells to respond to repeated peroxide exposure. When we investigated the ability of peroxide to stimulate ERK1/2 activation, we noted that with four successive peroxide exposures there was a significant reduction in the PC12 responsiveness to peroxide (Figures 3(c) and 3(d)). Interestingly, there seemed to be a specificity of the chemical sensitivity of the extant ERK1/2 response (R4) following the multiple stimuli compared to the initial response in naive cells (R1). Hence, for the R4 response point, the inhibitory effects of AG1478 preincubation upon peroxide-induced ERK1/2 activation were significantly diminished (Figure 4(d)). This reduced EGFR kinase dependence of the peroxide-induced R4 response was correlated to a significant reduction in the degree of peroxide-induced EGFR tyrosine phosphorylation (Figure 4(b)), attenuated peroxide-induced association of EGFR with Grb2 (Figures 5(a) and 5(d)), and a strong redistribution of EGFR away from the plasma membrane to the cytosol (Figures 6(e) and 6(i)). In contrast to this, the activation of Pyk2, its physical association with Grb2, and its subcellular localization were not significantly affected from the R1 to the R4 response point. This suggests that while peroxide stimulation can employ diverse tyrosine kinase scaffolds, the relative contribution of these to the signaling effects of peroxide is sensitive to the context of the oxidative stress. While we have focused on a limited number of downstream targets, it is highly likely that other effector outputs of peroxide stimulation could be differentially affected by repetitive exposures in a similar way to more classical “receptor-signaling” systems [38, 56–58]. The mechanisms by which oxidative stress and exposure are linked to ERK1/2 activity are clearly numerous and complex and therefore are probably subject to multiple levels of regulatory control. With respect to this, one primary stress-controlled ERK-regulatory mechanism may involve the MAPK phosphatases [MKPs: 59]. MKPs, which belong to the dual-specificity family of protein tyrosine phosphatases (DUSP), negatively regulate the phosphorylation and therefore activity status of kinases such as ERK1/2 [59, 60]. MKP-1, also known as DUSP1, is the prototypic member of the MKP family and has been demonstrated to become activated in response to oxidative stress [61, 62]. Through this regulatory capacity, it is not surprising that MKPs have been associated with cell growth, senescence and aging [63, 64]. Many cells therefore potentially possess a complex network of evolutionarily conserved response modalities linking oxidative stress and MAPK regulation. In future studies, we may yet further appreciate the true signaling depth and complexity of the chemically minimal signaling molecules such as hydrogen peroxide. In addition to this, the functional signaling capacity, which may include control of receptor or non-receptor transmembrane tyrosine phosphatases [65, 66], of hydrogen peroxide could be targeted to control oxidative stress responses in a similar manner that has been so successful for GPCRs.
